# Supplementary material for: Modeling glioblastoma heterogeneity as a dynamic network of cell states
Source: Mol Syst Biol. 2021 Sep 16;17(9):e10105. doi: 10.15252/msb.202010105 (PMC8444284; doi:10.15252/msb.202010105)
Supplement: Supplementary file 6 — Source Data for Figure 5 [file MSB-17-e10105-s004.zip › Figure5A_sourcedata/GSEA_3071/nosigdb_stateV.GseaPreranked.1622795159954/GANDAL_AUTISM_UP_PMID29439242.html]

Details for gene set GANDAL\_AUTISM\_UP\_PMID29439242[GSEA]

|  || Dataset | state43071 |
| Phenotype | NoPhenotypeAvailable |
| Upregulated in class | na\_neg |
| GeneSet | GANDAL\_AUTISM\_UP\_PMID29439242 |
| Enrichment Score (ES) | -0.56682 |
| Normalized Enrichment Score (NES) | -2.8173265 |
| Nominal p-value | 0.0 |
| FDR q-value | 0.0 |
| FWER p-Value | 0.0 |
Table: GSEA Results Summary

  

Fig 1: Enrichment plot: GANDAL\_AUTISM\_UP\_PMID29439242      
 Profile of the Running ES Score & Positions of GeneSet Members on the Rank Ordered List

  

| PROBE | GENE SYMBOL | GENE\_TITLE | RANK IN GENE LIST | RANK METRIC SCORE | RUNNING ES | CORE ENRICHMENT || 1 | HIST1H1C |  |  | 7 | 1.976 | 0.0356 | No |
| 2 | CRYAB |  |  | 176 | 1.221 | 0.0264 | No |
| 3 | CDKN1A |  |  | 363 | 0.993 | 0.0094 | No |
| 4 | STC1 |  |  | 433 | 0.920 | 0.0134 | No |
| 5 | ADAMTS9 |  |  | 726 | 0.733 | -0.0287 | No |
| 6 | KIF20A |  |  | 1133 | 0.583 | -0.0954 | No |
| 7 | PDLIM4 |  |  | 1382 | 0.511 | -0.1333 | No |
| 8 | UCP2 |  |  | 1779 | 0.422 | -0.2011 | No |
| 9 | FKBP5 |  |  | 1893 | 0.405 | -0.2151 | No |
| 10 | ANXA1 |  |  | 2165 | 0.366 | -0.2601 | No |
| 11 | SRPX |  |  | 2646 | 0.305 | -0.3461 | No |
| 12 | TIPARP |  |  | 2768 | 0.292 | -0.3638 | No |
| 13 | DNAJB1 |  |  | 3143 | 0.256 | -0.4305 | No |
| 14 | RHBDF2 |  |  | 3281 | -0.263 | -0.4518 | No |
| 15 | HMOX1 |  |  | 3426 | -0.286 | -0.4740 | No |
| 16 | LCP1 |  |  | 3470 | -0.294 | -0.4767 | No |
| 17 | HSPA1A |  |  | 3494 | -0.299 | -0.4755 | No |
| 18 | IFITM2 |  |  | 3585 | -0.312 | -0.4869 | No |
| 19 | DDIT4L |  |  | 3632 | -0.320 | -0.4897 | No |
| 20 | EEF1G |  |  | 3796 | -0.349 | -0.5143 | No |
| 21 | LY96 |  |  | 3829 | -0.354 | -0.5138 | No |
| 22 | YBX3 |  |  | 3863 | -0.361 | -0.5133 | No |
| 23 | TUBB6 |  |  | 3904 | -0.368 | -0.5141 | No |
| 24 | ITGB2 |  |  | 3948 | -0.377 | -0.5153 | No |
| 25 | PTTG1IP |  |  | 4002 | -0.389 | -0.5181 | No |
| 26 | YAP1 |  |  | 4041 | -0.398 | -0.5180 | No |
| 27 | DDIT4 |  |  | 4182 | -0.432 | -0.5366 | No |
| 28 | MTHFD2 |  |  | 4212 | -0.444 | -0.5339 | No |
| 29 | FAM129A |  |  | 4244 | -0.454 | -0.5313 | No |
| 30 | CYR61 |  |  | 4384 | -0.497 | -0.5486 | No |
| 31 | IFITM3 |  |  | 4424 | -0.509 | -0.5466 | No |
| 32 | TNFRSF10B |  |  | 4531 | -0.555 | -0.5564 | Yes |
| 33 | ITGA5 |  |  | 4580 | -0.576 | -0.5548 | Yes |
| 34 | TXNIP |  |  | 4618 | -0.604 | -0.5506 | Yes |
| 35 | TAGLN2 |  |  | 4648 | -0.618 | -0.5446 | Yes |
| 36 | SERPINB1 |  |  | 4659 | -0.623 | -0.5349 | Yes |
| 37 | P4HA1 |  |  | 4689 | -0.641 | -0.5284 | Yes |
| 38 | MSN |  |  | 4698 | -0.644 | -0.5179 | Yes |
| 39 | PIM1 |  |  | 4783 | -0.699 | -0.5209 | Yes |
| 40 | PALLD |  |  | 4839 | -0.749 | -0.5174 | Yes |
| 41 | DSE |  |  | 4903 | -0.818 | -0.5141 | Yes |
| 42 | TGM2 |  |  | 4928 | -0.842 | -0.5030 | Yes |
| 43 | ZFP36L1 |  |  | 4929 | -0.843 | -0.4872 | Yes |
| 44 | SLC16A3 |  |  | 4949 | -0.863 | -0.4747 | Yes |
| 45 | IGFBP5 |  |  | 4978 | -0.897 | -0.4633 | Yes |
| 46 | HSPB1 |  |  | 4992 | -0.920 | -0.4486 | Yes |
| 47 | COL8A1 |  |  | 5000 | -0.931 | -0.4325 | Yes |
| 48 | S100A10 |  |  | 5011 | -0.954 | -0.4165 | Yes |
| 49 | EMP1 |  |  | 5023 | -0.979 | -0.4003 | Yes |
| 50 | GBP1 |  |  | 5051 | -1.031 | -0.3862 | Yes |
| 51 | ANXA2 |  |  | 5055 | -1.036 | -0.3674 | Yes |
| 52 | SERPINH1 |  |  | 5059 | -1.050 | -0.3483 | Yes |
| 53 | ACKR3 |  |  | 5065 | -1.065 | -0.3294 | Yes |
| 54 | EMP3 |  |  | 5084 | -1.113 | -0.3120 | Yes |
| 55 | TIMP1 |  |  | 5134 | -1.251 | -0.2980 | Yes |
| 56 | TGFBI |  |  | 5136 | -1.259 | -0.2746 | Yes |
| 57 | GADD45B |  |  | 5155 | -1.335 | -0.2531 | Yes |
| 58 | S100A11 |  |  | 5196 | -1.536 | -0.2320 | Yes |
| 59 | RARRES3 |  |  | 5206 | -1.582 | -0.2041 | Yes |
| 60 | MYOF |  |  | 5207 | -1.590 | -0.1744 | Yes |
| 61 | NQO1 |  |  | 5210 | -1.596 | -0.1449 | Yes |
| 62 | TM4SF1 |  |  | 5235 | -1.812 | -0.1156 | Yes |
| 63 | MGP |  |  | 5252 | -2.037 | -0.0805 | Yes |
| 64 | COL4A1 |  |  | 5266 | -2.279 | -0.0404 | Yes |
| 65 | SPP1 |  |  | 5272 | -2.446 | 0.0044 | Yes |
Table: GSEA details [plain text format]

  

Fig 2: GANDAL\_AUTISM\_UP\_PMID29439242: Random ES distribution      
 Gene set null distribution of ES for **GANDAL\_AUTISM\_UP\_PMID29439242**
